# Grow fast, die young: Does compensatory growth reduce survival of juvenile blacktip sharks (*Carcharhinus limbatus*) in the western Gulf of Mexico?

**DOI:** 10.1002/ece3.8311

**Published:** 2021-11-10

**Authors:** Philip Matich, Jeffrey D. Plumlee, Mark Fisher

**Affiliations:** ^1^ Marine Biology Department Texas A & M University at Galveston Galveston Texas USA; ^2^ Institute of Marine Sciences University of North Carolina at Chapel Hill Morehead City North Carolina USA; ^3^ Rockport Marine Science Laboratory Coastal Fisheries Division Texas Parks and Wildlife Department Rockport Texas USA

**Keywords:** allometric growth, match/mismatch, natal site fidelity, parturition, shark nursery

## Abstract

Effective conservation and management necessitate an understanding of the ecological mechanisms that shape species life histories in order to predict how variability in natural and anthropogenic impacts will alter growth rates, recruitment, and survival. Among these mechanisms, the interaction between parturition timing and prey availability frequently influences offspring success, particularly when postnatal care is absent. Here, we assess how parturition timing and nursery conditions, including prey abundance and environmental conditions, influence the growth and potential survival of blacktip sharks (*Carcharhinus limbatus*) in western Gulf of Mexico (GOM) estuaries over their first year. Catch data from long‐term gillnet monitoring allowed for clear delineation of cohorts based on size frequency distribution plots, and showed that late parturition cohorts born in estuaries with fewer prey resources exhibited more rapid growth than early parturition cohorts that experienced more abundant prey. Compensatory behaviors that promoted accelerated growth led to reduced second year residency, likely due to reduced survival resultant from greater risk taking and potentially due to reduced site fidelity attributed to larger body size. Water temperatures influenced blacktip growth rates through physiological increases in metabolism and potential premigratory foraging cues associated with cooling temperatures. Gradual warming of the GOM (0.03°C year^−1^) was also correlated with earlier parturition across the study period (1982–2017), similar to other migratory species. Considering current trends in climate and associated phenological shifts in many animals, testing hypotheses assessing compensatory growth‐risk trade‐offs is important moving forward to predict changes in life histories and associated recruitment in concert with current and future conservation actions, like wildlife management.

## Graphical abstract

Late parturition, reduced prey abundance, and suboptimal temperatures led to compensatory growth of juvenile blacktips during their first year prior to winter emigration. Behaviors contributing to compensatory growth likely led to observed declines in first year survival. Warming Gulf of Mexico waters from 1982 to 2017 were correlated with earlier parturition, slower growth, and higher survival of age 0 blacktips.

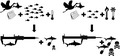

## 1 INTRODUCTION

Among the distinguishing characteristics of the 20th century, changes in wildlife management triggered by the extirpation and near extirpation of hundreds of wildlife populations have led to vast improvements in conservation practices (Evans, 2012; Hutton et al., 2005; Kennelly & Broadhurst, 2002). Yet, many populations and species are still imperiled by human actions, including sharks and their relatives, which are increasingly listed by CITES and the IUCN because of continued overharvesting, habitat deterioration, and fisheries bycatch (Dulvy et al., 2014). Low fecundity and late age of maturity limit population resilience and contribute to the challenges of managing many shark species (Dulvy et al., 2014). Concomitantly, sharks lack postparturition care and must contend with ecological risks while developing foraging skills without parental guidance or protection (reviewed in Hussey et al., 2010). Consequently, the conservation of juvenile shark populations is often a priority to ensure recruitment meets management targets (Kinney & Simpfendorfer, 2009). However, neonatal sharks innately avoid predators and seek out prey (Lyons et al., 2020) aided by their sociality (Guttridge, 2020), sensory biology and morphology (Gardiner et al., 2012), and nursery habitats, the latter of which have received significant attention because of their role in promoting juvenile shark survival (Heithaus, 2007).

Many shark nurseries occur in nearshore ecosystems that limit adult occurrence due to shallow depths and environmental conditions, thereby reducing predation risk (McCandless et al., 2007). While some bold individuals risk predation to access more metabolically rewarding habitats (e.g., Dhellemmes et al., 2020; Dibattista et al., 2007; Matich & Heithaus, 2015), most newborn sharks that use nurseries remain in these habitats for at least their first months of life, increasing the survival rate of young‐of‐the‐year (YOY, age 0) sharks (Heupel et al., 2007). The benefits derived from shark nurseries can be attributed to regional and natal philopatry exhibited by females of some species during optimal seasons for newborn growth and survival (Chapman et al., 2015; Hueter et al., 2005). Predictable parturition in low‐risk habitats during periods of high productivity expectantly increase individual fitness and in turn population resilience of some sharks. Thus, despite a lack of parental care after birth, the location and timing of parturition are investments made by pregnant sharks, with consequences for miscues considering the growth and survival of juveniles closely align with extrinsic factors like food availability and environmental conditions (Kerby et al., 2012; Rosa et al., 2014; Siddon et al., 2013; Wilson et al., 2021).

Intraspecific variability in behavior is common among sharks, and the timing and location of parturition expectantly varies among pregnant females that may lead to variability in litter and cohort success (e.g., Hoffmayer et al., 2013; Sen et al., 2018; Sulikowski et al., 2016). Delayed birth may lead to inadequate time for sharks to develop foraging skills and invest in somatic growth. Smaller sharks are more vulnerable to predators like larger sharks (Grubbs, 2010; Heithaus, 2004), which coupled with less time to develop antipredator behavior in seasonally constrained ecosystems, could lead to reduced offspring survival when parturition is mistimed (Visser & Gienapp, 2019). However, the costs of delayed parturition can be alleviated by several factors. Greater parental investment in offspring size or energy reserves can offset costs by reducing predation risk or food stress (reviewed in Kindsvater & Otto, 2014; Lyons et al., 2020; Pettersen et al., 2015). Similarly, compensatory growth can allow juvenile sharks to “catch‐up” to conspecifics born earlier, but inherent trade‐offs may affect survival (Hector & Nakagawa, 2012; Hornick et al., 2000). Increased growth rates require behavioral and/or physiological shifts, with an increase in food consumption and/or greater caloric allocation to structural size (Dmitriew, 2011). Animals that exhibit compensatory growth must:

1. increase time allocated to feeding, thereby reducing predator vigilance and/or time in refuge,
2. increase foraging rates that can reduce muscle quality and subsequent locomotor performance,
3. forage in more productive, but often riskier habitats, and/or
4. increase caloric contribution to structural growth, consequently reducing contributions to energetic reserves and increasing risk of environmental and food stress (Dmitriew, 2011).

Increased foraging has been hypothesized as a means by which species compensate for late parturition in other aquatic (e.g., Moginie & Shima, 2018), semiaquatic (e.g., Orizaola et al., 2010), and terrestrial species (e.g., Michel et al., 2018), but not without costs. For example, juvenile lemon shark (*Negaprion brevirostris*) growth rate in Bimini, The Bahamas was negatively correlated with survival, likely due to more frequent use of resource rich but risky foraging sites (Dhellemmes et al., 2020; Dibattista et al., 2007).

Although widely hypothesized, limited research has been conducted on compensatory growth in sharks due to the inherent challenges of studying wide ranging, highly mobile marine species that are often found in low abundances. In response to fishing pressure, juvenile Atlantic sharpnose sharks (*Rhizoprionodon terraenovae*) exhibited density‐dependent compensatory growth in the Gulf of Mexico (GOM), which led to earlier sexual maturation (Carlson & Baremore, 2003). Sandbar sharks (*Carcharhinus plumbeus*) exhibited a similar compensatory response to fishing pressure in the northwestern (NW) Atlantic (Romine et al., 2013; Sminkey & Musick, 1995), and NW Atlantic porbeagles (*Lamna nasus*) exhibited faster growth rates in response to fisheries harvesting (Cassoff et al., 2007). However, the compensatory response of porbeagles varied across age‐classes, with subadults growing faster and juveniles growing slower under exploitation (Cassoff et al., 2007). Comparatively, velvet belly latternsharks (*Etmopterus spinax*) in the northeastern Atlantic and Mediterranean (Coelho et al., 2010) and spiny dogfish (*Squalus acanthias*) in the NW Atlantic (Sosebee, 2005) exhibited smaller sizes at maturity in response to exploitation, but growth rates were unaffected by fishing pressure suggesting earlier age of maturation.

In light of these limited studies, it is unclear what triggers compensatory growth in sharks, and how this affects juvenile shark survival. Physiological mechanisms that increase growth rates should reduce predation risk based on body size (Heithaus, 2004), but could alter immune function, predator avoidance, and resistance to environmental stress, thereby reducing long‐term survival (Álvarez, 2011; Hector & Nakagawa, 2012). As such, understanding the factors that lead to compensatory growth in juvenile sharks and its resultant effects on survival are essential for predicting when cohorts and populations are at heightened risk.

Blacktip sharks (*Carcharhinus limbatus*) are among the most abundant shark species in the GOM, with a recent estimate of *ca*. 39 million individuals in 2016 (NMFS, 2018). GOM blacktips are born in spring–early summer at <50 cm total length (TL) in litter sizes of *ca*. 5 (Baremore & Passerotti, 2012) and are predicted to grow *ca*. 15 cm TL annually during their first few years before reaching sexual maturity at *ca*. 135 cm TL for females (Baremore & Passerotti, 2012; Deacy & Moncrief‐Cox, 2019). Like many other coastal sharks in the GOM, YOY blacktips use coastal estuaries as nurseries from Texas to Florida (McCandless, Kohler, et al., 2007). However, cooling water temperatures require blacktips to leave their nurseries during winter months, with at least some individuals exhibiting natal site fidelity (e.g., Hueter et al., 2005). Spatiotemporal and interindividual variability in reproductive biology, life history, and nursery conditions lead to variability in blacktip growth, behavior, and survival across nurseries and cohorts (Baremore & Passerotti, 2012; Carlson et al., 2006; Deacy & Moncrief‐Cox, 2019; Matich et al., 2021; McCandless, Kohler, et al., 2007). As such, blacktips provide an opportunity to assess the factors that lead to compensatory growth in juvenile sharks and how this affects survival.

We used standardized long‐term sampling from 1982 to 2017 across five Texas estuaries to test the following hypotheses:

- Later date of birth, reduced food availability, and suboptimal environmental conditions trigger compensatory growth among YOY blacktips.
- Conditions that lead to compensatory growth result in reduced survival of YOY blacktips during their first winter.

## 2 METHODS

### 2.1 Study area

**FIGURE 1:**
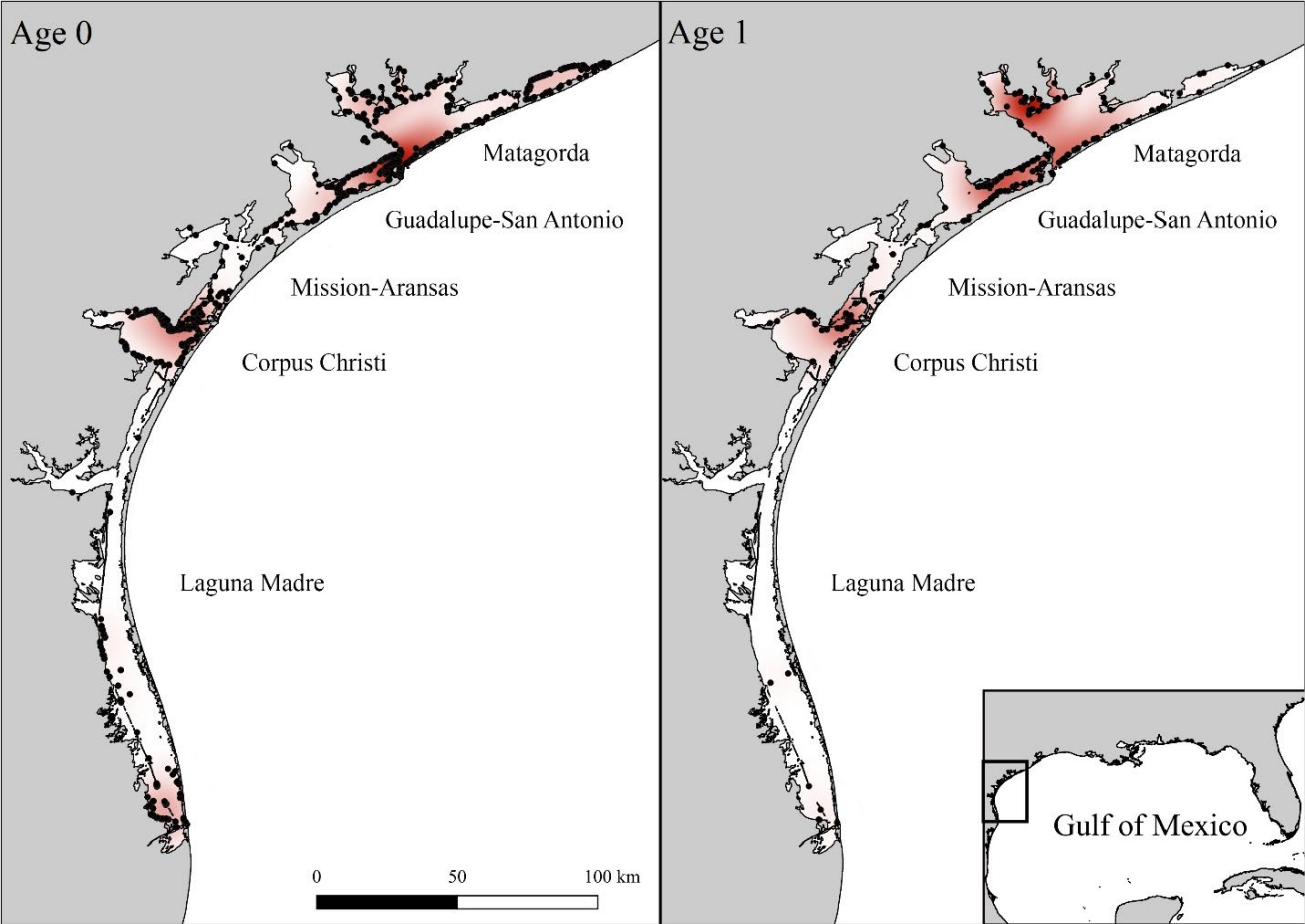
Central‐lower Texas coast along the western Gulf of Mexico. Black dots indicate capture locations of YOY (left panel) and age 1 (right panel) blacktips, with darker red coloration indicative of higher shark densities

The northwestern GOM includes more than 5000 km of Texas shoreline that expands across a series of estuaries, which serve as nurseries for juvenile sharks (Froeschke et al., 2010; Hueter & Tyminski, 2007; Swift & Portnoy, 2021; TinHan et al., 2020; Figure 1). The barrier islands that separate these estuaries from the GOM limit tidal inflow. As a result, most estuaries are brackish and experience spatiotemporal variability in freshwater inflow from connected river systems (Froeschke, Stunz, & Wildhaber, 2010; Longley, 1994; US EPA, 1999). Freshwater inflow from northeastern rivers is significantly greater than from southwestern rivers, resulting in hyposaline estuaries along the northeastern coastline (e.g., Sabine Lake) and hypersaline estuaries along the southwestern coastline (e.g., Laguna Madre; Longley, 1994; US EPA, 1999). Consequently, blacktips are very rarely found in Sabine Lake and exhibit variable abundances in Galveston Bay due to its dynamic low‐brackish salinity regimes (Plumlee et al., 2018). Previous research has also found differences in the population structure of other estuarine‐dependent juvenile carcharhindids (*C*. *leucas*) sampled in Sabine Lake and Galveston Bay when delineated from more southern Texas estuaries (TinHan et al., 2020). Therefore, neither Sabine Lake nor Galveston Bay were considered for the study to ensure that regional variability in population drivers did not confound the interpretation of results. Data from remaining estuaries (Figure 1) were pooled to assess regional patterns in juvenile blacktip compensatory growth in light of small sample sizes in some estuaries in some years.

### 2.2 Data collection

Species‐relative abundance and corresponding environmental data were obtained from standardized bag seine, otter trawl, and gillnet surveys conducted by the Texas Parks and Wildlife Department (TPWD) long‐term fishery‐independent monitoring program from 1982 to 2017. Within each estuary, 20 bag seines and 20 bay trawls were pulled monthly between dawn and dusk each year using a stratified cluster design within a 3.4225 km^2^ (1 nautical mile^2^) grid system. Bag seines (18.3 m long, 1.8 m deep with 1.3 cm stretched nylon monofilament mesh) were pulled parallel to the shoreline across an area of 0.03 hectares. Otter trawls (6.1 m wide with 38 mm stretched nylon multifilament) were pulled at 4.82 km/h for 10 min away from the shoreline in open water ≥1 m in depth.

Ninety gillnets per year were also set in each estuary using a stratified cluster design within the same 3.4225 km^2^ grid system in 10‐week spring (April–June; *n* = 45) and fall (September–November; *n* = 45) seasons. Monofilament gillnets (183 m long; 1.2 m deep with ascending 45.7 m sections of 7.6, 10.2, 12.7, and 15.2 cm stretched mesh) were set perpendicular to the shoreline at dusk and retrieved after dawn (mean soak time ± SD = 13.7 ± 1.4 h).

All organisms caught in seines, trawls, and gillnets were identified, counted, and measured (total length ‐ TL). Blacktips were only caught in gillnets during the study. Date, location, and water temperature (°C) were recorded for each sample. It is important to note that Laguna Madre was divided into two separate regions (upper and lower) and sampled as independent systems during monitoring.

Blacktips sampled with gillnets in San Antonio Bay in 2018 were also weighed to assess ontogenetic shifts in somatic growth (*n* = 40). Simple linear regression was used with power functions of mass:length for YOY and age 1 sharks separately to identify ontogenetic differences in isometric growth (expected mass:length) versus allometric growth (greater than or less than expected mass:length).

### 2.3 Cohort assessment

**FIGURE 2:**
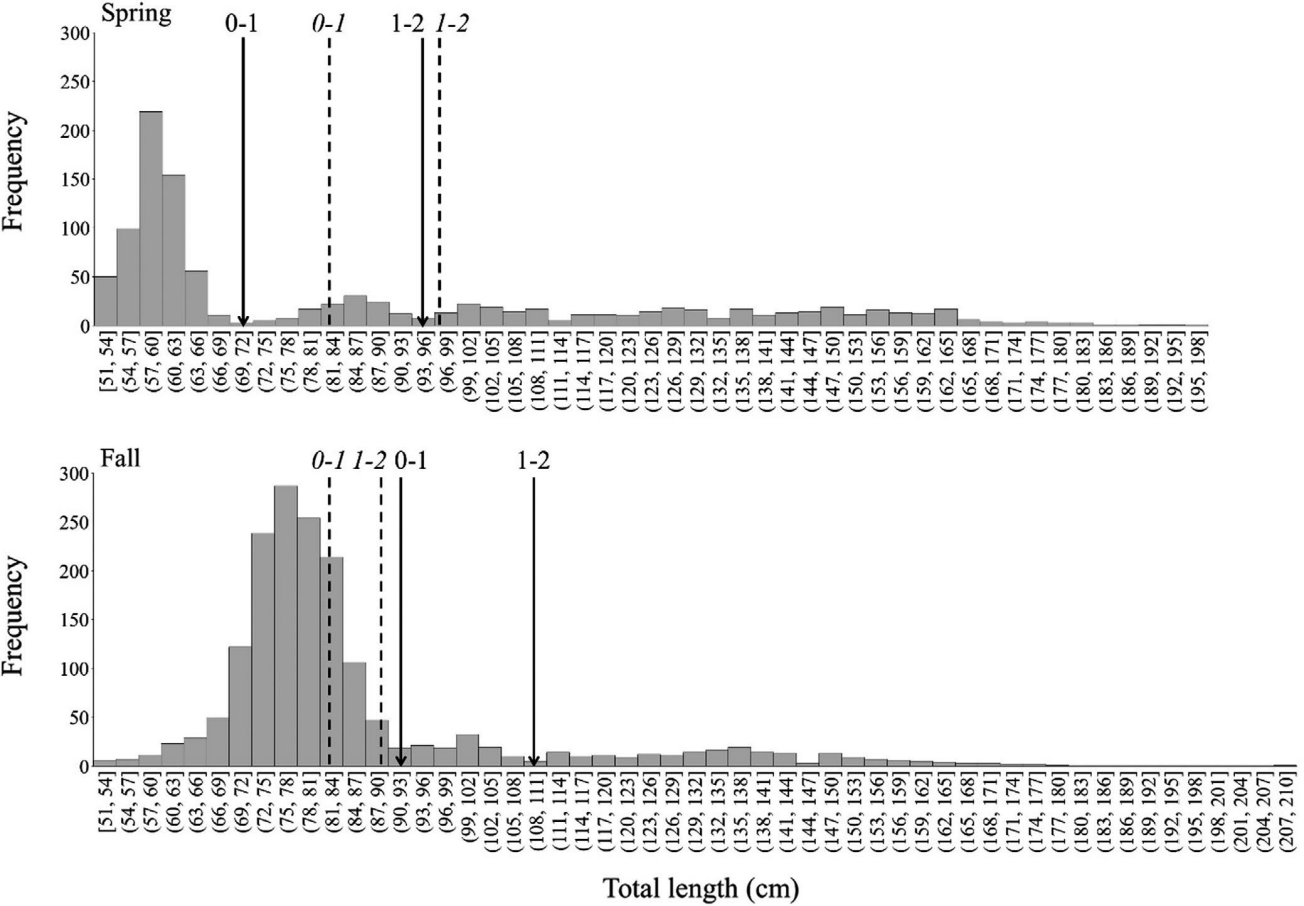
Size‐based frequency distribution of blacktips sampled in Spring (April–June) and Fall (September–November) across the study period (1982–2017). Dashed lines indicate estimated transition sizes from age 0 to age 1 (0–1) and age 1 to age 2 (1–2) based on published VBGFs for GOM blacktips (Deacy & Moncrief‐Cox, 2019). Solid lines indicate reassigned transition sizes from age 0 to age 1 and age 1 to age 2 based on the location of histogram valleys, which were used for analyses

The most recently published von Bertalanffy growth functions (VBGFs) for GOM blacktips (Deacy & Moncrief‐Cox, 2019) were initially used to assign age estimates of sharks based on shark TL, sampling date, and predicted parturition season (i.e., spring; Baremore & Passerotti, 2012). In order to assess the efficacy of assigned ages, size‐based frequency distribution histograms with 3‐cm TL bins were plotted for each season (Figure 2). Clear discrepancies with estimates based on VBGFs were found for the average transitions from age 0 to 1 and from age 1 to 2 based on histogram valleys, which may be attributed to geographic variability in juvenile blacktip growth rates and size at birth across the region (e.g., Carlson et al., 2006). As such, estimated age‐classes were reassigned. Sharks <70.6 cm TL in spring and <91.6 cm TL in fall were reassigned as YOY. Blacktips 70.6–94.5 cm TL in spring and 91.6–109.5 cm TL in fall were reassigned as age 1.

Monthly variability in YOY and age 1 blacktip abundances (sharks/gillnet) were then assessed with Kruskall‐Wallis tests to identify parturition periods (timing of first YOY blacktip occurrence), as well as the timing of emigration from and immigration to Texas estuaries in response to seasonal temperature shifts based on non‐normal data distributions (Froeschke, Stunz, & Wildhaber, 2010; Hueter et al., 2005). A similar assessment using Kruskall‐Wallis tests was used to identify monthly variability in the abundance of primary prey items for YOY blacktips (Gulf menhaden [*Brevoortia patronus*] based on bag seine data, and Atlantic croaker [*Micropogonias undulatus*] based on otter trawl data) to assess if parturition and immigration matched prey availability. Gulf menhaden and Atlantic croaker were chosen due to their predominance in the diets of YOY blacktips in the GOM (reviewed in Matich et al., 2021). Data were pooled across estuaries and years for monthly assessments, and post hoc Dunn's tests were used to identify significant differences between months.

In order to identify cohorts that exhibited compensatory growth, simple linear regression was used to estimate annual variability in growth rates from age 0 to 1 (first year growth) using the slope of best fit lines. Capture date (day of year [DOY]) was the independent variable, and blacktip TL was the dependent variable. Sharks were assigned to cohorts based on age estimates and capture dates (Matich et al., 2020). YOY blacktips were sampled in spring every year of the study with the exception of 1996, which was removed from analyses to avoid spurious results attributed to this cohort.

Simple linear regression was also used to assess the impacts of YOY abundance and compensatory growth on second year residency (i.e., site fidelity to Texas estuaries during age 0–1 transition). Annual deviations from mean estimated growth rate was the independent variable, and the ratio of age 1:age 0 sharks in the same cohort was the dependent variable to account for potential bias attributed to annual variability in cohort size.

### 2.4 Generalized additive models

To investigate the influence of the environment on interannual differences in first year growth rate, we used generalized additive models (GAMs), which are semiparametric analogs of generalized linear models that allow for nonlinear relationships between predictor and response variables (Wood, 2006). Models were run using a gaussian distribution and a logarithm link, and also run unconstrained using the default value for *k* in the mgcv package in R (Wood, 2017). The general GAM construction was

$$E\left[y\right]=g^{‐1}\left[\beta_{0}+\sum_{k}S_{k}\left(X_{k}\right)\right],$$

where *E*[y] is equal to the expected value of the response variable (catch), *g* is the link function, *β_0_* is the intercept, *X* represents one of *k* predictor variables, and *S_k_* is the smoothing function of the predictor variable *X_k_* (Wood, 2006).

To assess environmental and ecological factors that may affect first year growth, fifteen predictor variables were identified as candidate covariates for the model, including water temperature, salinity regime, prey availability, competition, adult fecundity, and parturition timing. Water temperature measurements collected from TPWD bag seine surveys were used to define five potential covariates: mean total temperature, mean seasonal temperature (spring, summer, and fall), and the number of days with a measured temperature of >34.6°C (95th percentile) per annum across the study area, which may affect growth through physiological changes in metabolic demands (Huey & Stevenson, 1979). Mean annual winter (December 1–March 31) sea surface temperature (SST) measurements were also collected from waters over the continental shelf in the northwestern GOM (N 27.125°, W 93.875°) using NOAA’s Advanced Very High Resolution Radiometer Pathfinder version 5.3 L3‐Collated SST (measurement accuracy of 0.05°/5 km; https://coastwatch.pfeg.noaa.gov/), which could affect first year over‐wintering success (e.g., Brodersen et al., 2011; Djurichkovic et al., 2019).

Factors affecting estuarine salinity regimes, including freshwater inflow and El Niño and La Niña weather patterns, were also included as potential covariates (Froeschke, Stunz, & Wildhaber, 2010). Archival data of annual river discharge measurements were collected from 13 available USGS monitoring stations at the headwaters of Texas estuaries that collected data for the duration of the study period (1982–2017) ranging north to south from N 29.309°, W 96.104° to 27.711°, W 97.502° (https://dashboard.waterdata.usgs.gov/). The presence/absence of El Niño and La Niña were based on NOAA’s Oceanic Niño Index that assesses anomalies in SST based on a 30‐year average (https://origin.cpc.ncep.noaa.gov/products/analysis_monitoring/ensostuff/ONI_v5.php).

To identify potential covariates pertaining to prey availability, mean annual catch per unit effort (CPUE) of Gulf menhaden from bag seine surveys and mean annual CPUE of Atlantic croaker from otter trawl surveys were used as estimates of relative abundance of prey. Potential competition covariates included annual blacktip and bull shark (*Carcharhinus leucas*) CPUE from gillnet surveys (Cottrant et al., 2021; Matich et al., 2021). Adult fecundity was assessed as a potential covariate based on published annual spawning stock size from the most recent GOM blacktip stock assessment (NMFS, 2018). Annual parturition timing was estimated from the first occurrence of a YOY blacktip sampled in gillnets.

Manual stepwise backwards variable selection minimizing Akaike information criterion (AIC) was used to optimize variable contribution to the model where only significant (*p* < .05) variables were retained within the model. If correlations among variables were identified (*r* > .2), correlated variables were tested individually using separate models (with correlations typically occurring within like‐variables; e.g., temperature, freshwater inflow, and shark biomass), and the variable that was attributed to a better model fit (lowest AIC and highest Deviance Explained [DE]) was used in the final model. This procedure resulted in multiple models given the combination of correlated covariates, with the final, most parsimonious model (significant variables and lowest AIC) selected from all of the unique combination of covariates. Upon final model selection for growth rate, independent variables retained in the growth rate GAM were also employed to assess predictability of second year residency across sampling years using a second GAM.

## 3 RESULTS

From 1982 to 2017, more than 2700 YOY (annual mean ± SE; 51.9 ± 8.8) and age 1 (9.8 ± 1.7) blacktips were caught, with cohorts ranging from 20 to 241 sampled individuals (Table 1). As expected, YOY sharks were caught in spring (April–June) across all years, except for 1996 (removed from analyses), and in fall (September–November) across all years.

**TABLE 1:** Cohort variability in the relative abundance of age 0 and age 1 blacktips, slope (cm TL/day) and test statistics for best fit lines, and estimated first year growth is based on the slope of best fit lines (in cm TL)

Cohort	*n* (age 0)	*n* (age 1)	DOY 1st age 0	Slope	*r* ^2^	*F*	*p*	Estimated 1st year growth
1982	25	2	127	0.101	.69	50.2	<.001	36.8
1983	26	2	145	0.110	.78	86.9	<.001	40.0
1984	14	8	130	0.065	.66	23.6	<.001	23.7
1985	29	38	127	0.115	.76	87.5	<.001	42.0
1986	236	5	149	0.097	.35	125.1	<.001	35.2
1987	39	21	133	0.069	.71	88.6	<.001	25.0
1988	127	23	123	0.086	.69	284.1	<.001	31.5
1989	96	11	109	0.092	.66	184.3	<.001	33.5
1990	54	2	143	0.113	.58	72.7	<.001	41.3
1991	101	1	141	0.142	.82	447.8	<.001	51.7
1992	18	2	133	0.077	.70	37.6	<.001	28.3
1993	43	11	139	0.085	.76	126.9	<.001	31.1
1994	56	9	151	0.094	.67	108.5	<.001	34.4
1995	31	11	129	0.072	.62	47.4	<.001	26.1
1997	28	3	161	0.170	.92	316.2	<.001	62.2
1998	25	12	148	0.121	.61	35.8	<.001	44.0
1999	72	21	117	0.061	.61	109.9	<.001	22.1
2000	49	10	122	0.089	.74	134.3	<.001	32.6
2001	104	5	128	0.042	.24	31.9	<.001	15.4
2002	39	14	141	0.085	.66	71.2	<.001	31.1
2003	129	15	140	0.085	.71	304.2	<.001	30.9
2004	37	23	131	0.102	.64	63.1	<.001	37.2
2005	58	19	130	0.078	.70	130.7	<.001	28.6
2006	43	6	144	0.080	.42	29.1	<.001	29.3
2007	29	11	128	0.062	.70	63.1	<.001	22.6
2008	53	8	119	0.083	.80	203.3	<.001	30.3
2009	30	9	132	0.091	.74	79.7	<.001	33.4
2010	17	9	138	0.062	.62	24.9	<.001	22.6
2011	60	28	130	0.077	.85	321.7	<.001	28.2
2012	96	31	143	0.053	.73	250.6	<.001	19.2
2013	192	18	128	0.084	.73	519.9	<.001	30.7
2014	67	5	149	0.064	.44	51.5	<.001	23.3
2015	44	34	104	0.048	.39	27.3	<.001	17.4
2016	87	3	131	0.114	.79	321.8	<.001	41.5
2017	168	NA	109	0.064	.79	629.2	<.001	23.5

**FIGURE 3:**
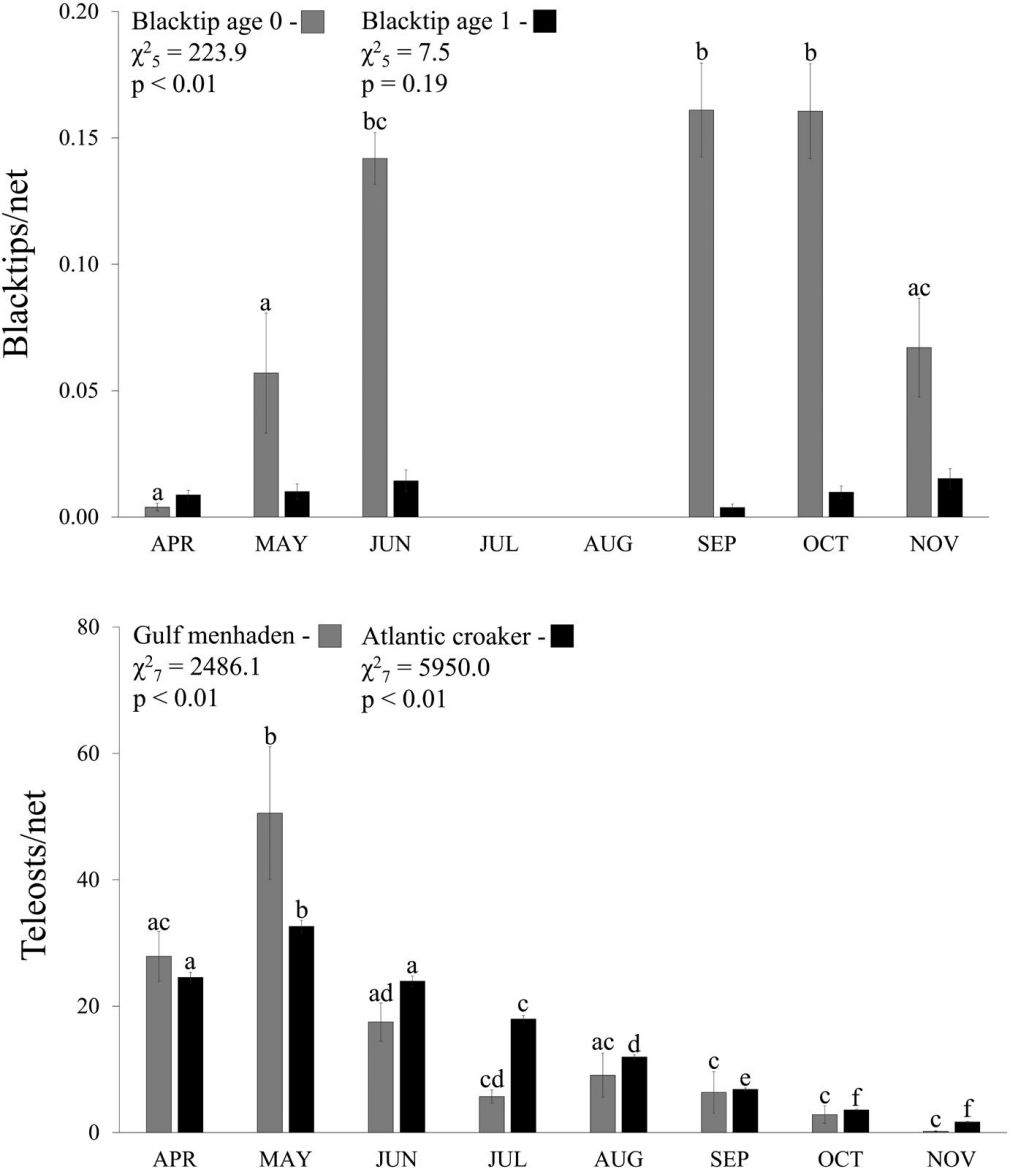
Relative abundances of age 0 and age 1 blacktips based on gillnet sampling, and primary YOY prey (Gulf menhaden [bag seine sampling] and Atlantic croaker [otter trawl sampling]). Primary prey were sampled across all months, whereas blacktips were not sampled in July and August. Error bars are ±SE, and letters above error bars indicate significant differences the relative abundances of shark or prey based on post hoc analysis

The relative abundance of YOY sharks exhibited monthly variability, with a significant increase from April–May to June indicative of parturition and a significant decrease from October to November indicative of emigration (Figure 3). The primary prey species of YOY blacktips (i.e., Gulf menhaden and Atlantic croaker) also varied by month (Figure 3). The highest relative abundances of both prey species was in May matching hypothesized blacktip parturition, with significant decreases through November (Figure 3). Comparably, age 1 blacktips exhibited no monthly variability in relative abundance (Figure 3). Blacktips from San Antonio Bay in 2018 exhibited an ontogenetic shift in mass:length, with YOY sharks exhibiting slightly negative allometric growth (*b* = 2.67) indicative of greater energetic allocation to length than mass, and age 1 sharks exhibiting substantial positive allometric growth (*b* = 5.00) indicative of greater energetic allocation to mass than length (Appendix S1).

**FIGURE 4:**
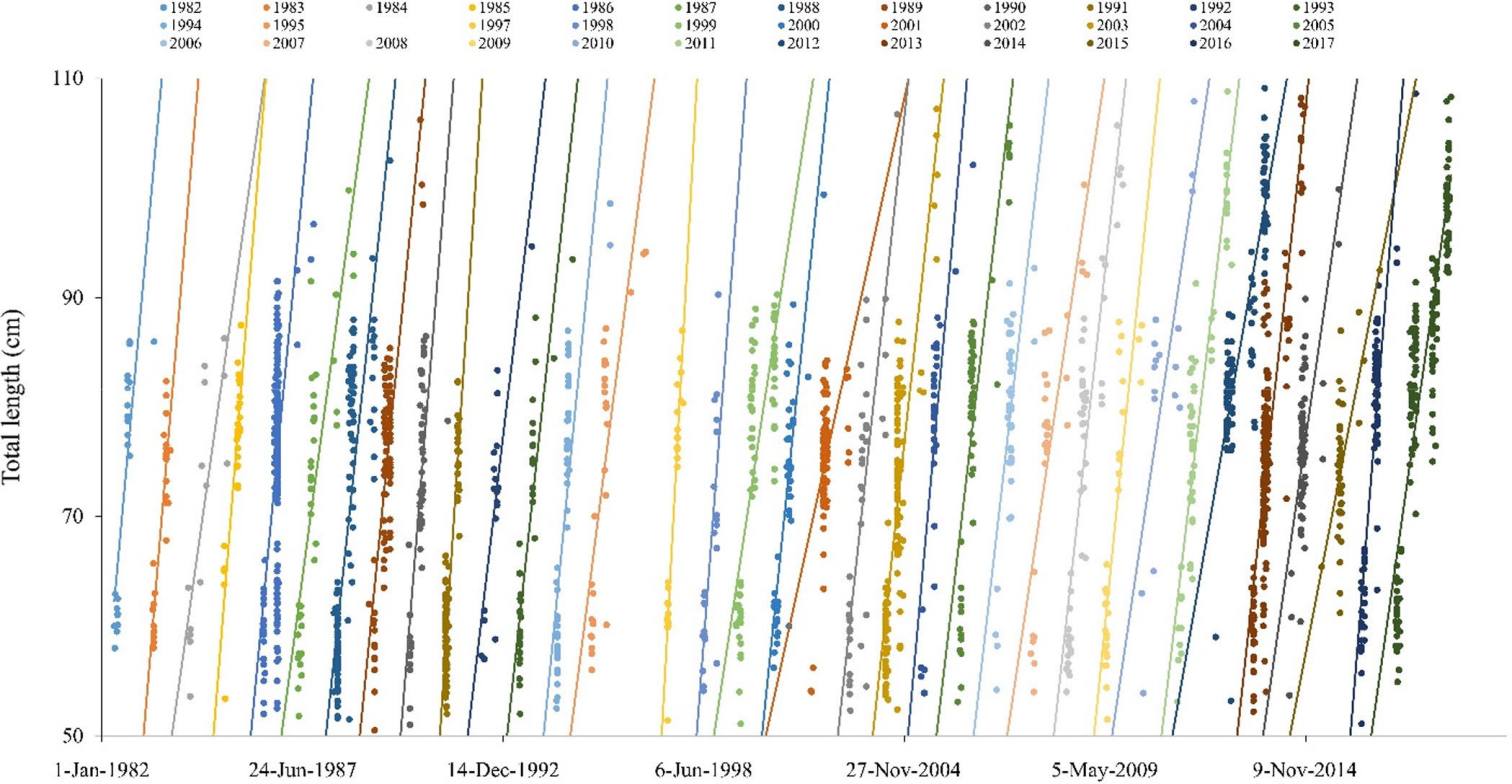
Total length upon capture date used to estimate first year growth of blacktip cohorts based on shark age estimates (see Figure 2). Best fit lines are from linear regressions, and colors delineate different cohorts. No age 0 sharks were sampled in Spring 1996; thus, the 1996 cohort was removed from analyses

**FIGURE 5:**
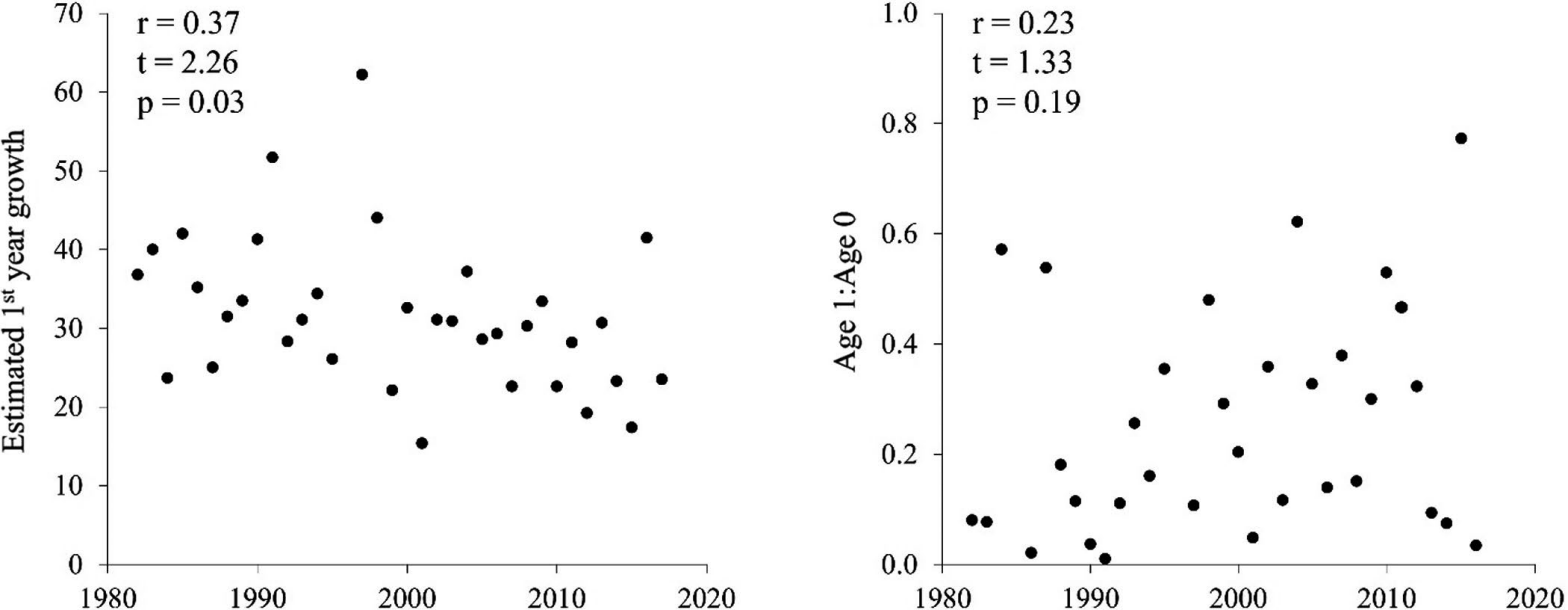
Annual trends in estimated first year growth and second year residency (age 1:age 0 blacktips), with correlation test statistics and *p*‐values

**FIGURE 6:**
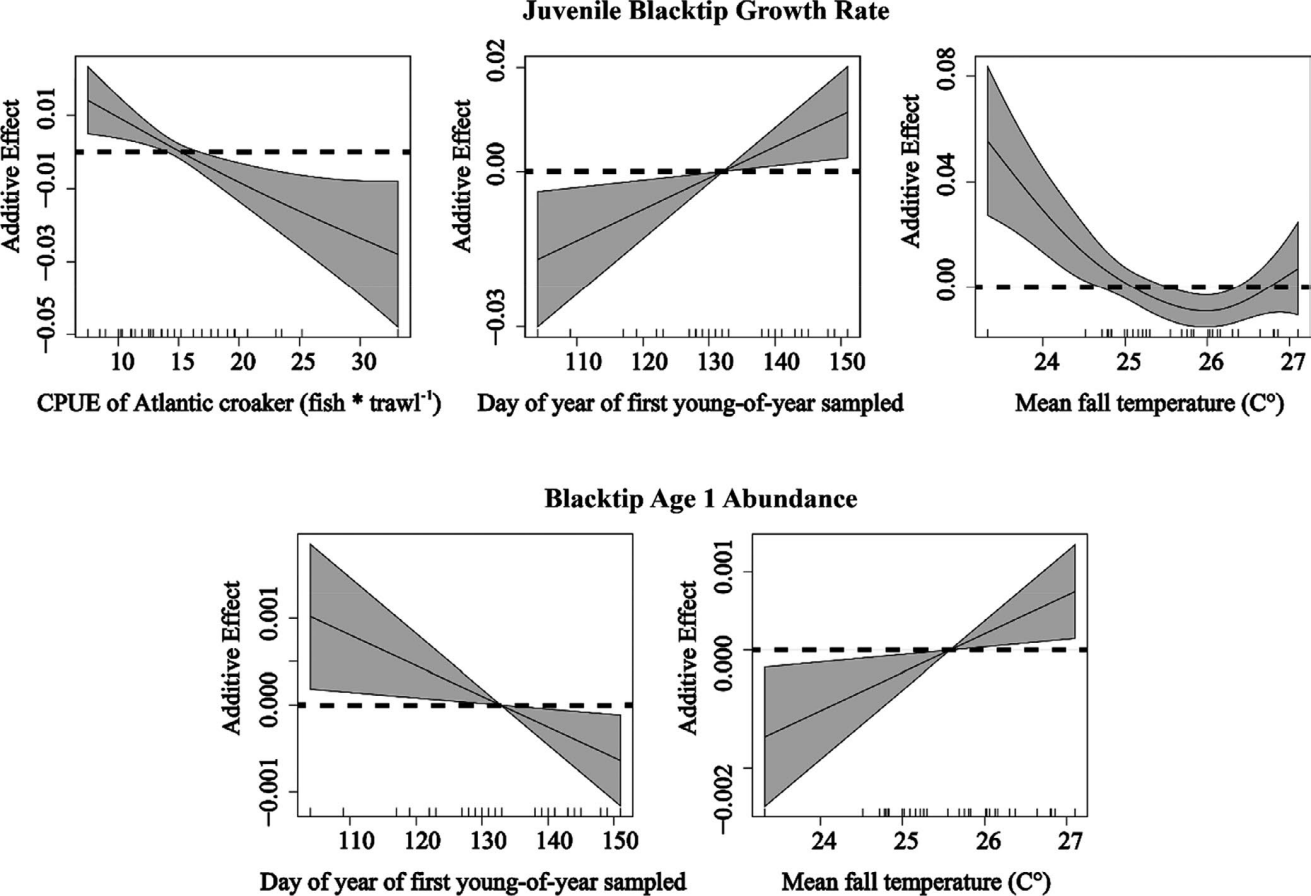
Generalized additive model (GAM) response plots showing the influence of retained variables on the juvenile growth rate and standardized abundance of age 1 blacktips, including CPUE of Atlantic croaker (fish * trawl^−1^), the mean fall water temperature (°C), and day of year of first YOY sampled

Cohorts exhibited considerable differences in the date YOY blacktips were first sampled (April 14–June 10; mean = May 13; Table 1), as well as estimated growth rates based on the slopes of best fit lines (Figure 4), both of which generally decreased from 1982 to 2017 (Figure 5). The final, most parsimonious GAM for growth rate included mean fall water temperature (edf = 1.21, *F* = 7.71, *p* < .05), day of first collection (i.e., parturition estimate; edf = 1.00, *F* = 6.76, *p* < .05), and CPUE of Atlantic croaker (edf = 2.70, *F* = 5.88, *p* < .05), with 57.9% of deviance explained (Table 2; Figure 6).

**TABLE 2:** Abiotic and biotic variables retained in the final GAMs after stepwise backwards AIC selection process using growth rate and standardized age 1 abundance as dependent variables

	Juvenile Blacktip growth rate			Age 1 abundance		
Model	AIC	DE		AIC	DE	
	−174.2	57.9%		−351.9	31%	

	∆AIC	∆DE	Estimated threshold	∆AIC	∆DE	Estimated threshold
CPUE of Atlantic croaker	9.8	21.2	15 fish * trawl^−1^	–	–	
Day of year of first young‐of‐year sampled	5.3	10.0	132.5 day of year	4.2	14.2	132.5 day of year
Mean fall temperature (°C)	13.8	34.0	25.0°C	4	14.8	25.6°C

Note

Model suitability was interpreted from AIC scores and percent deviance explained (DE%). The relative importance of each variable was estimated given the difference in AIC (∆AIC) and DE (∆DE) when this variable was removed from the final model.

**FIGURE 7:**
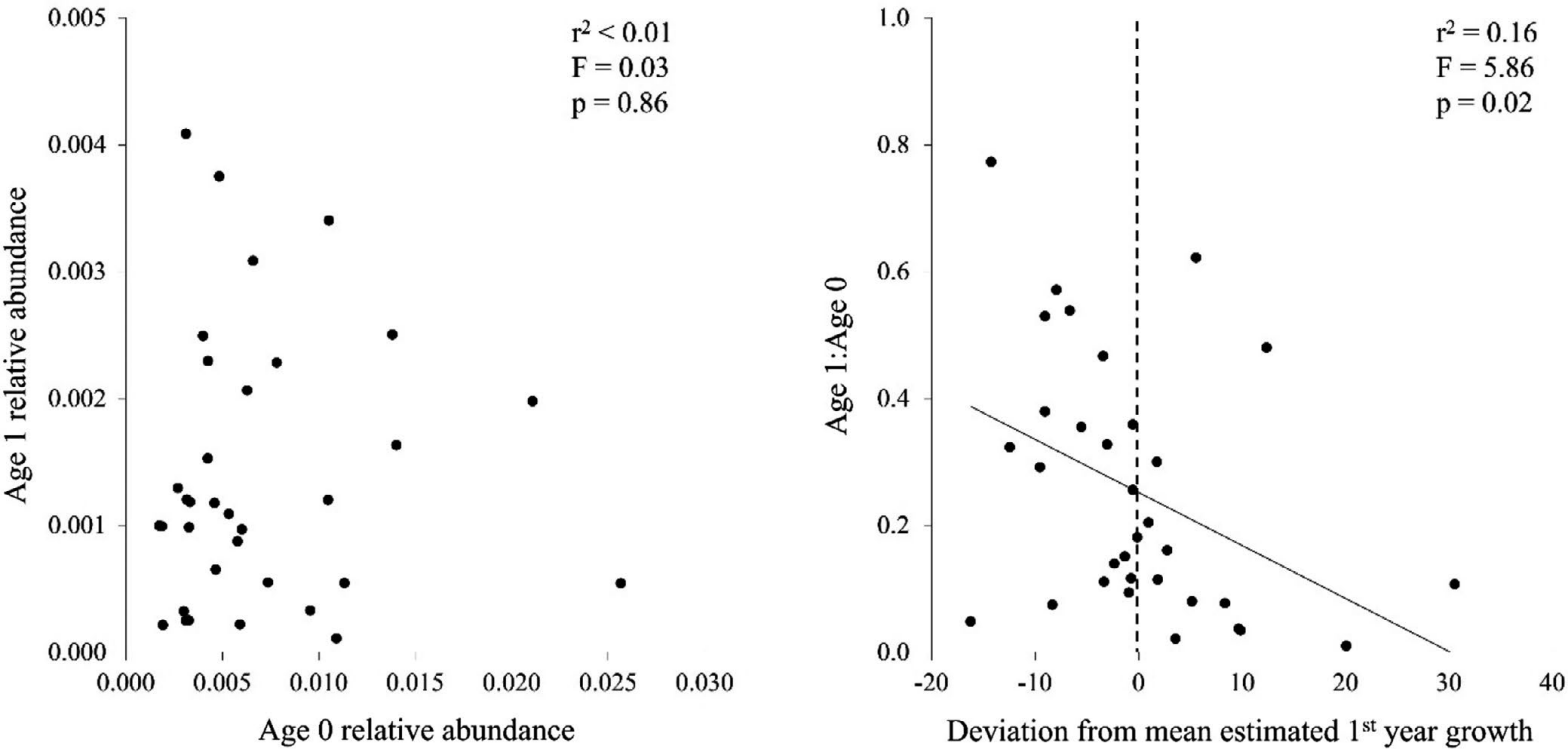
Linear relationships between the relative abundances of age 0 and age 1 blacktips (left) and deviations from mean estimated first year growth (based on cohort slopes described in Table 1 and Figure 4), and the ratio of age 1:age 0 within cohorts (right). Dashed line in right panel indicates location of mean predicted first year growth, which intersects best fit line at *ca*. 0.25 age1:age0

The abundance of age 1 sharks exhibited no significant linear relationship with YOY abundance in the same cohort (Figure 7). However, the standardized abundances of age 1 sharks (age 1:age 0 for each cohort) exhibited a significantly negative relationship with first year growth, with an average second year residency of *ca*. 0.25 at mean growth indicating that on average *ca*. 25% of YOY blacktips returned to Texas estuaries after their first winter, assuming no sampling mortality (Figure 7). Standardized abundances of age 1 sharks exhibited a positive, but nonsignificant correlation with sampling year (Figure 5). The final, most parsimonious GAM using the standardized abundances of age 1 sharks as the dependent variable built on the results from the growth rate GAM and included mean fall temperature (edf = 1.00, *F* = 6.20, *p* < .05) and day of first YOY collection (edf = 1.00, *F* = 5.95, *p* < .05) with 31% of deviance explained (Table 2; Figure 6).

## 4 DISCUSSION

Among the conservation challenges of the 20th century, many shark species experienced near extirpation across large portions of their geographic ranges (Dulvy et al., 2014). In turn, the implementation of fisheries management plans in some countries, and collaborative international efforts to reduce harvesting, bycatch, and habitat deterioration coupled with an enhanced understanding of shark biology and ecology have improved the status of some populations (Carlson et al., 2019). Yet, human actions are not the only threat to reproliferation. Natural variability in biotic and abiotic factors play key roles in shark abundance (e.g., Drymon et al., 2013; Dudley & Cliff, 2010; Plumlee et al., 2018), and the ecological conditions juvenile sharks encounter play pivotal roles in their growth and survival during early life history stages (Heithaus, 2007). YOY blacktip cohorts that experienced fewer prey, warmer or cooler water temperatures than average, and reduced time in estuaries prior to winter emigration exhibited compensatory growth, which may have reduced first year survival compared to cohorts born earlier, and in years with more abundant prey populations and moderate temperatures.

Like many other sharks in subtropical and temperate latitudes, juvenile blacktips in the GOM migrate to more equatorial waters along continental shelves in fall–early winter to avoid cooling nearshore waters, and may return in spring–early summer to further utilize the nursery functions of coastal estuaries (e.g., Hueter et al., 2005; Logan et al., 2020; Reyier et al., 2014). Larger body size energetically and ecologically aids in migratory behavior (e.g., Acolas et al., 2012; Nasby‐Lucas et al., 2019; Zhao et al., 2018). Thus, longer developmental time in nurseries and/or compensatory growth should provide benefits for juvenile sharks preparing for winter emigration and potential postwinter fidelity to nursery habitats (Hueter et al., 2005; Ulrich et al., 2007). Rapid first year growth across many shark species supports this hypothesis (Cailliet & Goldman, 2004). Based on presence/absence data, the average estuarine departure date for the last YOY blacktips sampled within the study area was October 29. As such, YOY blacktips had up to *ca*. 6 months on average to refine foraging and antipredator behavior, and allocate energy to structural growth and energy reserves within Texas estuaries, comparable to sharks in other estuaries across the region (McCandless, Kohler, et al., 2007).

Yet, intraspecific variability is pervasive across sharks, including emigration and parturition dates (e.g., Hoffmayer et al., 2013; McCandless et al., 2007; Sulikowski et al., 2016), which may lead to variability in litter and cohort success (Visser & Gienapp, 2019). The average date when YOY blacktips first occurred in Texas gillnets (May 13) fits predicted parturition timing (Baremore & Passerotti, 2012), and was used as an estimate of when parturition began in the study area. However, first YOY occurrence ranged from April 14 to June 10 during the study, and estuarine departure date for the last YOY blacktip ranged from September 22 to November 19. Consequently, late parturition cohorts had up to 40% less first year developmental time in estuaries compared to early parturition cohorts (4.1 and 6.9 total months, respectively), with 49% of variability in first year residence time explained by predicted parturition initiation. As such, parturition timing may be a key factor in first year success. For example, delayed hatching led to significantly smaller body size and lower fledging success of European great tit (*Parus major*) and blue tit (*P*. *caeruleus*) chicks resultant from mistimed phenology (abundance, quality) of primary prey species (*Operophtera bumata*; Buse et al., 1999). Fishes (e.g., Durant et al., 2005), mammals (e.g., Plard et al., 2014), and invertebrates (e.g., Visser & Both, 2005) also exhibit negative effects when parturition is mistimed with food availability. Prey populations (i.e., Atlantic croaker and Gulf menhaden) peaked in May across Texas estuaries. Thus, mismatched blacktip parturition coupled with less time in estuaries after parturition could reduce foraging opportunities for some cohorts and subsequently affect growth, development, and survival before and during winter emigration (Visser & Gienapp, 2019).

Delayed parturition was, however, not fatal for all sharks. Despite missing peak prey abundance, late parturition cohorts exhibited accelerated first year growth rates, suggesting that food resources are not limiting for YOY blacktips and other predators in Texas estuaries. Compensatory growth previously documented in other shark populations resulted from declines in abundance, with subsequent density‐dependence release promoting increased growth rates (e.g., Carlson & Baremore, 2003; Romine et al., 2013). Comparatively, first year growth among blacktips in Texas was not influenced by shark abundance, and reductions in prey populations led to increased first year growth rates, supporting our hypothesis that food is not limiting. Additionally, regional differences in the body condition of juvenile bull sharks suggest that southern Texas estuaries are more productive than northern estuaries and those in the eastern GOM, providing further support (Garcia Barcia et al., 2021). Some mammals (e.g., *Odocoileus virginianus*; Michel et al., 2018), birds (*Cerorhinca monocerata*; Hirose et al., 2012), fish (e.g., *Forsterygion lapillum*; Moginie & Shima, 2018), amphibians (e.g., *Rana arvalis*; Orizaola et al., 2010), and invertebrates (e.g., *Pararge aegeria*; Nylin et al., 1989) that experience delayed birth also exhibit more rapid growth than earlier born conspecifics, providing fitness benefits associated with larger body size (Roff, 1992; Stearns, 1992). As such, our results add to studies indicating late‐born individuals can catch‐up through behavioral and/or physiological compensatory mechanisms (Hector & Nakagawa, 2012).

Late parturition, reduced prey abundance, and average fall water temperatures below *ca*. 25°C and above *ca*. 27°C were drivers of increased YOY growth rates. Beyond warmer water temperatures leading to increased metabolic activity and thus growth (Huey & Stevenson, 1979), other predictor variables suggest fast growing blacktip cohorts foraged more frequent and/or more efficiently to increase energy acquisition (Dmitriew, 2011). Juvenile sharks in other nurseries also exhibit intraspecific variation in foraging to improve metabolic status. For example, all juvenile bull sharks in a Florida estuary seasonally increased foraging in low risk, low salinity habitats to access allochthonous prey resources (Matich & Heithaus, 2014), but only some individuals foraged in high risk, high reward marine habitats the remainder of the year (Matich et al., 2011). Similarly, juvenile lemon sharks more willing to explore novel habitats in Bimini, The Bahamas, exhibited faster growth, but lower survival rates than less exploratory conspecifics, presumably in response to using more rewarding but riskier seagrass habitats (Dhellemmes et al., 2020). Thus, reduced food availability due to less abundant prey populations and late parturition may have led to increased foraging rates and/or larger search areas among YOY blacktips. Average fall water temperatures below *ca*. 25°C may have also served as a cue for YOY sharks to increase foraging rates/efficiency, and therefore risk taking in preparation of early winter migrations into the GOM (Matich & Heithaus, 2015).

As a result, the trade‐offs associated with prolonged compensatory behaviors, including reduced time in refuge and reduced vigilance during foraging, could have reduced survival (Dmitriew, 2011). Many YOY sharks face food‐risk trade‐offs, and often use small home ranges and low risk habitats to avoid encounters with potential predators (e.g., Heupel et al., 2004; Legare et al., 2018; Morrissey & Gruber, 1993). Consequently, compensatory growth poses a risk for YOY blacktips if rewarding but risky behaviors increase overlap with potential predators like large sharks in habitats proximate to the GOM (Lofthus, 2019; Matich & Heithaus, 2015; Werner & Gilliam, 1984). Blacktips born earlier in years with more abundant prey resources, and optimal fall water temperatures likely exhibited more conservative foraging behavior that led to reduce growth rates but increased survival, which is supported by patterns in second year (i.e., age 1) residency.

Site fidelity is exhibited by some juvenile sharks, including blacktips, which use nurseries during their first few years for the protective benefits these habitats provide (Chapman et al., 2015). While untested, several estuaries in the western GOM appear to be important blacktip nurseries based on repeated annual abundances of YOY sharks in the Matagorda and Guadalupe‐San Antonio estuaries, and the confluence of the Corpus Christi and Mission‐Aransas estuaries (Heupel et al., 2007). Based on the relative abundances of YOY and age 1 individuals, and assuming blacktips leave estuaries in late fall–early winter (Hueter et al., 2005; Parsons & Hoffmayer, 2007; Steiner et al., 2007), blacktips exhibited a *ca*. 25% first year return rate to the study area, which is comparable to other regions where blacktips exhibit site fidelity (e.g., Ulrich et al., 2007). However, second year residency was highly variable across cohorts during the study period (1–77%). While the duration of first year (YOY) residency was not correlated with second year residency, first year growth rate was (*r* = −.40), indicating faster growing cohorts were comprised of fewer sharks that returned to Texas estuaries. If compensatory growth requires risky behavior, then second year residency may serve as an indicator for first year survival, with YOY blacktips attempting to catch up in size exhibiting higher rates of mortality (Dhellemmes et al., 2020; Lima & Dill, 1990; Werner & Anholt, 1993). As such, YOY blacktips may overcome late parturition and suboptimal nursery conditions through compensatory growth, but the associated behaviors that increase energetic acquisition likely reduce first year survival, particularly if they persist in higher risk GOM waters during overwintering (Dmitriew, 2011).

Alternatively, compensatory growth may reduce the need for blacktips to return to Texas estuaries after their first year. Some migratory YOY blacktip populations permanently emigrate from natal nurseries (e.g., Gurshin, 2007; Hueter et al., 2007; Steiner et al., 2007), and Texas blacktips could have immigrated to more equatorial estuaries in Mexico proximate to overwintering waters (e.g., Rio Soto La Marina, San Andrés, Laguna de Tamiahua; Hueter et al., 2007, McCandless, Pratt, et al., 2007). However, size frequency distributions indicate that at least some YOY blacktips return to Texas estuaries like other juvenile populations in the region (e.g., Hueter & Tyminski, 2007; Parsons & Hoffmayer, 2007; Ulrich et al., 2007). Juvenile sharks exhibit ontogenetic shifts in home ranges (size and location) to meet growing metabolic needs (Grubbs, 2010), which could account for reduced second year residency among fast growing blacktip cohorts. Consequently, second year residency may be indicative of the speed of ontogenetic shifts in habitat use rather than survival, with a greater proportion of blacktips in fast‐growing cohorts spending a single season in natal estuaries. Yet, sampling data indicate a nearly equal proportion of age 0–1 (second year; 12% of sampled sharks) and age 1–2 sharks (third year; 9% of sampled sharks) caught in spring gillnets, suggesting blacktips use Texas estuaries for multiple years, and intercohort variability in second year residency is more likely attributed to survival than ontogenetic habitat shifts. Elegantly designed tracking and life history studies are needed to fully address this question though.

### 4.1 Caveats

While data analyses and interpretation fit within previous frameworks and ecological theory (Dhellemmes et al., 2020; Dmitriew, 2011; Matich et al., 2020), the assumptions of our study should be considered prior to drawing conclusions. Despite careful assignation of ages to blacktips, it is likely that some individuals were misclassified considering differences in in situ data and VBGFs (Figure 2), and inherent variability in birth sizes and growth rates within cohorts. Assigning a YOY shark as age 1 or vice versa would have implications in cohort assignation and in turn cohort growth rates and second year residency. While misclassification cannot be reconciled based on available data, the size structure of juvenile blacktips in the western GOM shows distinct cohort structuring from which to delineate age 0 and 1 sharks (Figure 2). As such, the vast majority of sharks were likely assigned correctly as YOY or age 1, with exceptions for YOY sharks larger than expected and age 1 sharks smaller than expected based on birth size or individual growth rate, with a higher likelihood of misclassification for age 1 than age 0 based on assignation criteria. Bias was likely equal across years based on the use of a priori criteria rather than post hoc visual identification, and the distinction of highly significant regression models for each cohort support the classification methodology.

An additional consideration is the use of the dates of first YOY and last YOY sampled as estimates of parturition initiation and emigration completion. While parturition estimates fit with predictions based on previous studies and comparable nurseries (Baremore & Passerotti, 2012; Hueter & Tyminski, 2007), parturition could have been earlier that April or later than June, and thus undetected during the spring sampling period. Indeed, age 0 sharks were sampled in the first week of spring (1989, 2015, 2017), and thus may have been present prior to sampling. Age 0 sharks were also undetected during spring sampling in 1 year (1996). However, these events were rare (8% and 3%, respectively), and 1996 was removed from analysis eliminating this confounding factor. Thus, the observed trends are unlikely attributed to the restricted spring sampling period, and the first occurrence of YOY blacktips in gillnets is likely a good estimate for the initiation of blacktip parturition in the western GOM.

Coordinated parturition is also unlikely (McCandless, Kohler, et al., 2007), though short parturition periods are exhibited by some populations (Castro, 2009), and the ecological benefits of synchronous parturition are evident considering large sharks (e.g., *Carcharhinus brevipinna*, *C*. *leucas*, *C*. *limbatus*) are present within Texas estuaries April–November (Ims, 1990; Lofthus, 2019; TPWD unpublished data). Interpretation should therefore be made at the cohort level rather than the litter or individual level—the ecological processes discussed apply to individual sharks and shark litters, but the consequences cannot be assessed at these organizational levels due to sampling constraints. Similarly, juvenile sharks rarely exhibit coordinated emigration beyond responses to extreme events (Huepel et al., 2003; McCandless, Kohler, et al., 2007; Strickland et al., 2019), thus estimated emigration dates were used for estimates of first year residency. Some YOYs were sampled in the last week of fall sampling (34% of years). Thus, final emigration dates could be later than estimated, particularly in warmer, more equatorial estuaries (e.g., Laguna Madre), and warrants further investigation, though emigration timing is comparable to that exhibited by YOY blacktips in the eastern GOM at similar latitudes (Hueter et al., 2005). Patterns are also unlikely to be uniform across estuaries. Sample sizes necessitated pooling data, providing regional rather than estuary/nursery‐specific patterns, but more refined studies should be considered in estuaries with elevated juvenile blacktip densities (i.e., Matagorda, Guadalupe‐San Antonio, and Corpus Christi estuaries).

## 5 CONCLUSIONS

While assessing the abundances and size structures of target species are among the primary aims for fisheries monitoring, the value of such programs reach beyond traditional stock assessments. Across the western GOM, long‐term monitoring by TPWD has provided insight into shark nursery function (Froeshcke et al., 2010), responses to environmental variability (Plumlee et al., 2018), and predator–prey relationships (Cottrant et al., 2021; Livernois et al., 2021). Our study illustrates the ecological mechanisms that shape variability in juvenile blacktip growth rates and residency patterns, and how monitoring parturition timing and water temperature can provide reliable predictions of early blacktip life history within the western GOM.

It is unclear what determines blacktip parturition timing in the western GOM. However, its gradually earlier occurrence during the study period (*ca*. 0.2 days earlier per year) could be attributed to warming GOM waters (*ca*. 0.03°C year^−1^) that reduce gestation time among blacktips, and in turn increase estuarine developmental time for YOY blacktips prior to winter emigration (Schlaff et al., 2014). Warming waters could lead to physiologically suboptimal nursery conditions that counteract early parturition benefits (Huey & Stevenson, 1979; Lyons et al., 2020). Yet, YOY blacktips exhibited a negative trend in growth rates, and a positive trend in second year residency during the study period (Figure 5), suggesting thermal thresholds have not yet been reached despite the *ca*. 0.05°C year^−1^ increase in average water temperatures from Matagorda estuary to Laguna Madre since 1982.

Despite the caveats discussed, our study provides a framework to test for compensatory growth‐risk trade‐offs across other species and ecosystems where long‐term monitoring is conducted moving forward (e.g., Benavides et al., 2021; Drymon et al., 2010). GOM blacktips are among the most economically important shark stocks in the region; thus, the implications of our study are of value for conservation and management (NMFS, 2018). However, assessments of more vulnerable species that use the GOM for nursery habitats (e.g., Hueter & Tyminski, 2007; Scharer et al., 2017) provide even greater promise moving forward considering current trends in sea level rise and warming water temperatures, and the associated phenological and ecological shifts observed among migratory species (Scranton & Amarasekare, 2017). Long‐term monitoring has been heralded as an integral aspect to ecology (Alber et al., 2013), and our study serves as one more reminder why such work should be supported.

## CONFLICT OF INTEREST

None declared.

## AUTHOR CONTRIBUTION

**Philip Matich:** Conceptualization (lead); Data curation (supporting); Formal analysis (equal); Investigation (lead); Methodology (lead); Project administration (lead); Supervision (lead); Visualization (lead); Writing‐original draft (lead); Writing‐review & editing (lead). **Jeffrey D Plumlee:** Conceptualization (supporting); Data curation (supporting); Formal analysis (equal); Investigation (supporting); Methodology (supporting); Visualization (supporting); Writing‐original draft (supporting); Writing‐review & editing (supporting). **Mark Fisher:** Data curation (lead); Resources (lead).

## Supporting information

Appendix S1

## Data Availability

Data are managed, archived, and made available by TPWD Coastal Fisheries (https://tpwd.texas.gov/about/administration‐divisions/coastal‐fisheries).
