# Supplementary material for: Grow fast, die young: Does compensatory growth reduce survival of juvenile blacktip sharks (Carcharhinus limbatus) in the western Gulf of Mexico?
Source: Ecol Evol. 2021 Nov 10;11(22):16280–95. doi: 10.1002/ece3.8311 (PMC8601900; doi:10.1002/ece3.8311)

Appendix 1: Mass:length of age 0 blacktips (gray; negative allometric growth) and age 1 blacktips (black; positive allometric growth) sampled in San Antonio Bay in 2018.


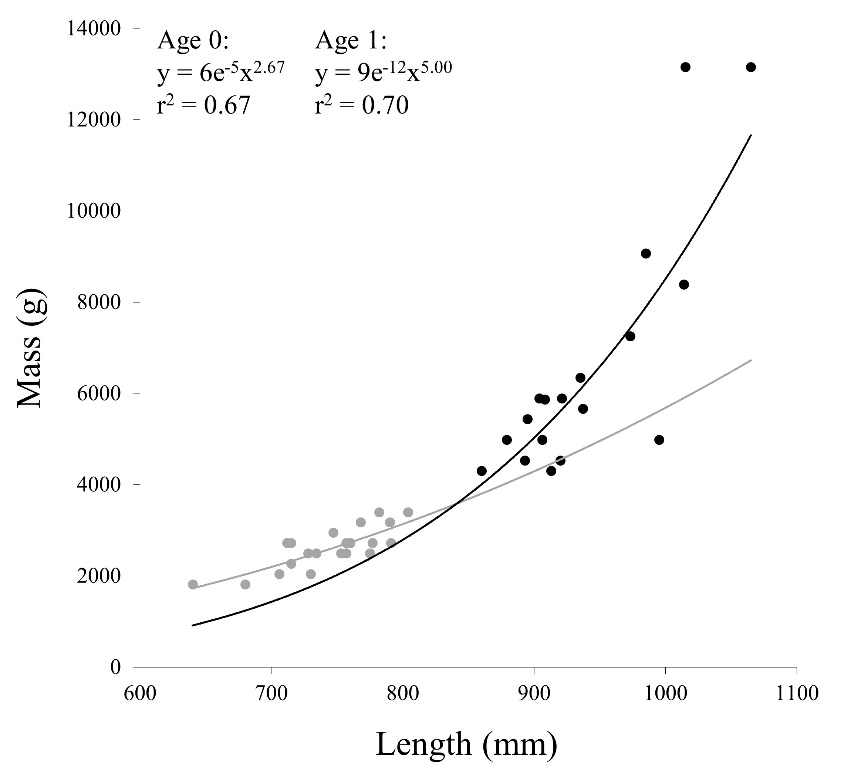

Supplement: Supplementary file 1 — Appendix S1 [file ECE3-11-16280-s001.docx]
